# Inclusion body fibromatosis

**DOI:** 10.4322/acr.2023.462

**Published:** 2023-12-15

**Authors:** Elgiva Iangngap, Mayur Parkhi, Aravind Sekar, Parmod Kumar, Uma Nahar Saikia

**Affiliations:** 1 Post Graduate Institute and Medical Education and Research, Department of Histopathology, Chandigarh, India; 2 Post Graduate Institute and Medical Education and Research, Department of Plastic Surgery, Chandigarh, India

**Keywords:** Toe Phalanges, Fibroma, Inclusion Bodies, Pathology, Microscopy, Electron

Inclusion body fibromatosis is a benign, often locally recurring myofibroblastic tumor with distinctive intracytoplasmic eosinophilic inclusions. In 1965, Reye^1^ was the first to document this entity through a series of 6 cases of digital fibrous tumors of childhood, where he observed intracytoplasmic inclusion bodies. This entity was later termed Reye’s tumor.^2^ As per the recent 2020 World Health Organization (WHO) classification, the other accepted terminologies include infantile digital fibroma/fibromatosis, recurring digital fibrous tumor(s) of childhood, and recurring digital fibroma(s) of childhood/infancy, where the etiology and pathogenesis remain unclear.^3^ It is a rare and slow-growing tumor that usually affects infants and children (mostly in less than 5 years) with equal gender distribution.^4^ These lesions most commonly affect the lateral and dorsal aspects of the last four digits, sparing the thumb, hand, or foot. Rarely, extremities, tongue, and breast act as the primary extradigital site. Grossly, the lesion appeared firm to rubbery and polypoidal dermal nodule with intact overlying stretched-out skin surface. Microscopically, the tumor cells contain characteristic intracytoplasmic 1.5 to 24 μm, rounded, pale pink round bodies located mainly in para nuclear location, highlighted better on Masson Trichrome (red color), phosphotungstic acid-hematoxylin (dark purple color), and Movat (pink color) stains. Ultrastructurally, the inclusions correspond to localized collections of non-membrane-bound, granular fibrillary material contiguous with the rough endoplasmic reticulum. These inclusions show strong immunoreactivity with actin and anti-calponin-1 antibody (inclusion containing calponin 1 as its possible content) but an occasional weak expression for caldesmon (the reason is still unknown). The recurrence rate is common with incomplete excision.^5^

**Figure 1:**
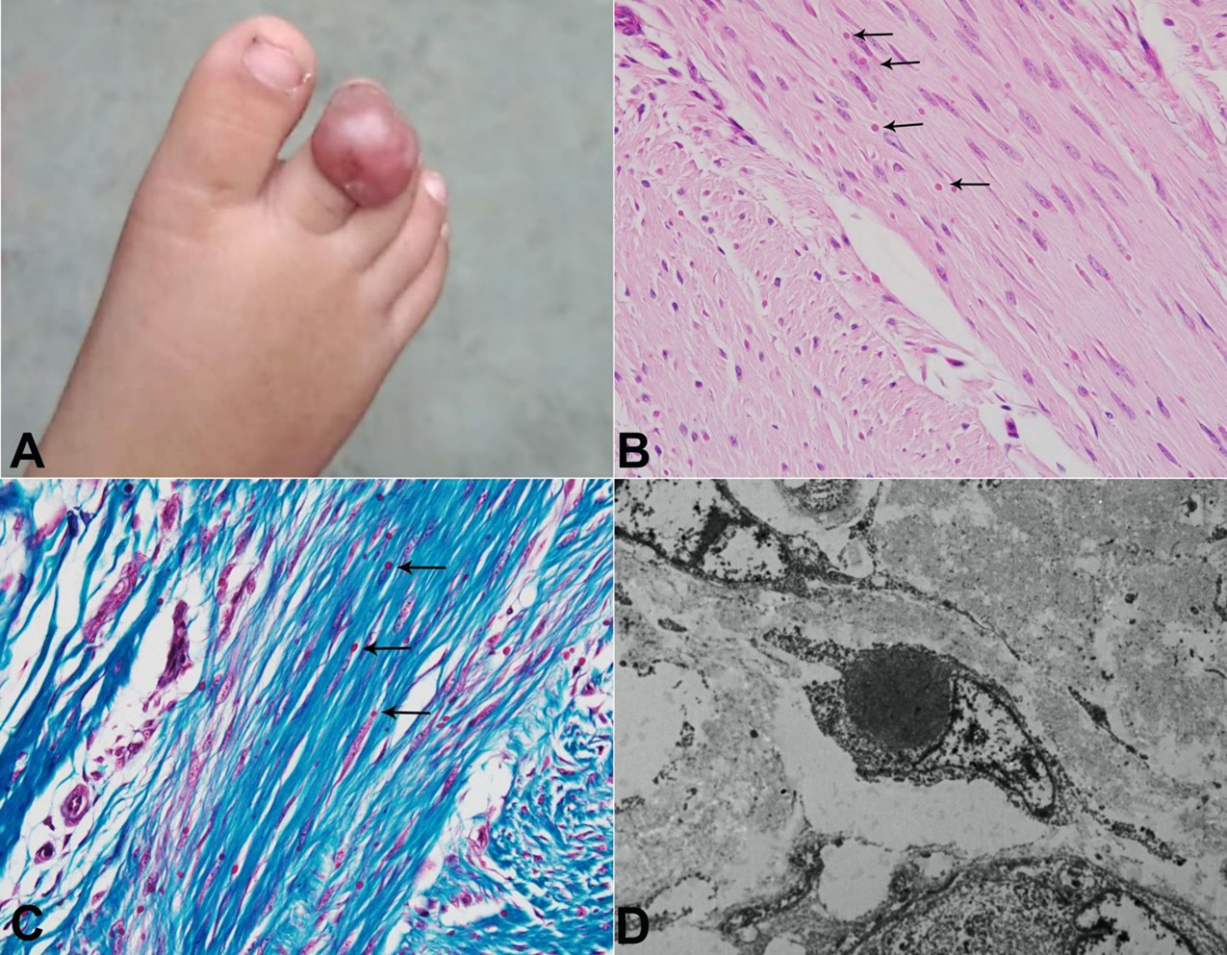
Inclusion body fibromatosis. **A -** a nodular swelling over the dorsal aspect of the proximal phalanx of the right second toe measuring 1.6x1.4cm in size (scale bar = 1 cm). The overlying skin was intact, erythematous, and stretched out; **B -** the spindle-shaped uniform tumor cells show many intracytoplasmic and para-nuclear eosinophilic bodies causing nuclear indentation of one of the poles at places- black arrows (H&E; x200); **C -** Masson Trichrome stain highlights these inclusions as red-coloured – black arrows (x200); **D -** ultrastructurally, the tumor cell within the cytoplasm shows a round, dense body (size: 3.12 µm) located in the para-nuclear region with corresponding nuclear indentation.

Figure 1 refers to a 2-year-old female child presented with nodular swelling over the dorsal aspect of the proximal phalanx of the right second toe (Figure 1A). The swelling appeared firm, slightly mobile, and non-tender, with a history of gradual increase in size since birth. The overlying skin was erythematous and stretched out. The mass was seen in close approximation with the nail bed. The respective interphalangeal and metatarsophalangeal joints were mobile. The plastic surgeons kept the possibility of fibromatosis and performed a Ray amputation of the right second toe. The specimen was submitted for histopathological examination.

The specimen grossly included a dislocated right second toe comprising proximal, middle, and distal phalanges, measuring 5.5cm in length and 1cm in width. In close proximity to the nail bed, the dorsal aspect showed a nodular mass measuring 1.6x1.3x0.4cm. The cut surface revealed a relatively well-demarcated lesion that was firm, homogenous, and white. The lesion was not reaching the underlying bone and also the surgical margins. Microscopically, the dermis showed a relatively well-circumscribed tumor with hyperkeratosis, parakeratosis, and flattened rete ridges of the overlying epidermis. The tumor was composed of spindle-shaped cells arranged in intersecting short fascicles and, at places, in whorls. These cells are benign and uniform and contain elongated nuclei, bland chromatin, inconspicuous nucleoli, and pale eosinophilic cytoplasm with indistinct cell membrane. Mitotic activity is infrequent (less than 1/10hpf) with no significant nuclear atypia or necrosis. In addition, many intracytoplasmic and para-nuclear eosinophilic bodies causing nuclear indentation of one of the poles at places were noted (Figure 1B). These inclusions appeared red-colored on the Masson Trichrome stain (Figure 1C), magenta-colored on the Periodic Acid-Schiff stain, and showed weak-to-moderate immunoreactivity for calponin immunostain. The surgical margins and the underlying bone were not involved in the tumor. Taking into account the clinical findings and histopathological aspects, a diagnosis of inclusion body fibromatosis was given. Subsequently, tissue was retrieved from a representative formalin-fixed, paraffin-embedded block and subjected to transmission electron microscopy. Ultrastructurally, the tumor cells showed round, dense bodies of varying sizes (2.97 µm to 4.32 µm) located in the para-nuclear region within the cytoplasm with corresponding nuclear indentation (Figure 1D). The tumor cells also contained bundles of myofilaments. Thus, the characteristic ultrastructural features further confirmed the diagnosis. Postoperatively, the patient was kept on close observation with no adjuvant therapy. At 4 months follow-up, no local recurrence was evident.
